# Genomic Insights into Historical Adaptation of Three Key Fungal Plant Pathogens

**DOI:** 10.1093/gbe/evaf241

**Published:** 2025-12-11

**Authors:** Joris A Alkemade, Edgar L Y Wong, Alan G Buddie, Matthew J Ryan, Timothy G Barraclough

**Affiliations:** Department of Biology, University of Oxford, Oxford, UK; Calleva Centre, Magdalen College, Oxford, UK; Department of Biology, University of Oxford, Oxford, UK; Senckenberg Biodiversity and Climate Research Centre, Frankfurt am Main, Germany; CAB International (CABI), Ascot, UK; CAB International (CABI), Ascot, UK; Department of Biology, University of Oxford, Oxford, UK; Calleva Centre, Magdalen College, Oxford, UK

**Keywords:** fungal evolution, fungicide resistance, population genetics, *Verticillium*, *Botrytis*, *Fusarium*

## Abstract

Fungal culture collections hold a wealth of historical isolates that could be used to study fungal evolution over the past decades, an era that coincided with agricultural industrialization. We performed population structure and temporal association analysis on three major fungal crop pathogens, *Verticillium nonalfalfae*, *Fusarium culmorum*, and *Botrytis cinerea*, collected between 1956 and 2023. Population structure analysis indicated predominantly sexual reproduction in *F. culmorum* and *B. cinerea*, whereas *V. nonalfalfae* was shown to be largely asexual. Single nucleotide polymorphisms (SNPs) of the recombining species *F. culmorum* and *B. cinerea* that showed major temporal changes fell within or close to coding genes, whereas time-variant SNPs in *V. nonalfalfae* were located within or close to transposable elements (TEs) and a *Starship* element. This is consistent with the hypothesis that rare-sex fungal species often rely on TE-mediated genomic diversification rather than sexual recombination. Across all three species, rapidly evolving SNPs were associated with genes encoding Major Facilitator Superfamily transporters, which are frequently implicated in fungicide resistance, and Zn2Cys6 fungal-type transcription factors, which play key roles in stress responses and pathogenesis. Our findings demonstrate the value of temporal association analysis as an untargeted approach for exploring fungal evolution since the advent of the green revolution. Applying this method across a broader range of fungal crop pathogens could provide deeper insights into their evolution and adaptation.

Significance Fungal pathogens are a big threat to global food production and are difficult to control due to their quick adaptation. By studying genomes of historical isolates of three major fungal plant pathogens collected over 70 years, we found that fungi use different evolutionary strategies to adapt: some rely on sexual reproduction to generate change, while others seem to depend on transposable elements. We identified genes linked to fungicide resistance and stress responses as frequent sites of adaptation, highlighting how genomic studies of historical collections can provide powerful insights into the ongoing evolution of crop pathogens.

## Introduction

Since the 1950s, the use of monocultures, pesticides, and artificial fertilizers have dramatically increased to improve productivity and limit land use expansion (Balmford et al. 2018). While this approach has been effective in maintaining food security, it has also caused severe environmental impacts and exerted strong selection pressure on crop pathogens. Today, fungal pathogens alone cause an estimated $220 billion in global crop losses annually (FAOSTAT, 2021). Management relies heavily on fungicides and resistance breeding, yet the adaptive capacity of these pathogens continue to undermine these efforts (Alkemade et al. 2025). This has led to the widespread emergence of fungicide resistance and repeated breakdown of host resistance genes (McDonald and Linde 2002; Lucas et al. 2015; Hawkins et al. 2019; Beckerman et al. 2023). For instance, the use of single-site fungicides since the 1970s selected for resistance via nonsynonymous point mutations in target genes, which reduce fungicide binding while maintaining pathogen fitness (Hawkins and Fraaije 2016; Fisher et al. 2018; Hawkins et al. 2019). Likewise, protection provided by single plant resistance genes is often rapidly overcome, sometimes within only a few years of field use, due to strong directional selection favoring virulent pathogen genotypes (McDonald and Linde 2002). This ongoing evolutionary arms race underscores the urgent need to better understand the mechanisms and dynamics of pathogen adaptation to design more durable and sustainable disease management strategies.

Historical fungal isolates preserved in culture collections and genome databases offer valuable resources for tracking pathogen evolution (Ryan et al. 2024). For example, historical isolates of *Fusarium xylarioides* spanning 52 years revealed horizontal gene transfer and shifts in effector composition contributing to new outbreaks (Peck et al. 2021, 2024). Analysis of a global *Zymoseptoria tritici* population collected between 1986 and 2016, identified mutations in CYP51 conferring resistance to azole fungicides (Feurtey et al. 2023). Similarly, analysis of 32 UK isolates from three fungal species (1950 to 2000) identified genomic regions under strong selection for both fungicide resistance and virulence (Wong et al., Submitted). These findings underscore the power of historical genomics to reveal evolutionary responses to agricultural practices. However, the utility of culture collections is often limited by unsystematic sampling or the lack of metadata on geography, host, or time. Despite this, their temporal and taxonomic range can yield insights that complement modern genomic studies.

Here we explore the evolution of three key fungal crop pathogens, *Fusarium culmorum*, *Verticillium nonalfalfae*, and *Botrytis cinerea*, using population genetics and temporal analyses. We applied binomial regression and genome wide association studies (GWAS), of historical isolates from the CABI culture collection (Ryan et al. 2024) and all publicly available genomes. The three species were selected based on their availability at CABI and agricultural importance. *F. culmorum*, a major causal agent of foot and root rot and Fusarium head blight (FHB) in mostly cereals, is a soil-borne pathogen with a strong saprophytic phase (Scherm et al. 2013). Though no sexual morph is known, some populations show signs of recombination (Miedaner et al. 2013). FHB has been widely controlled by demethylation inhibitor (DMI) fungicides, but resistance is emerging (Hellin et al. 2018). *V. nonalfalfae*, previously grouped with *V. albo-atrum* (Inderbitzin et al. 2011), is a soil-borne wilting pathogen affecting important crops such as hops, potato, and tomato. Little is known about its sexual cycle but the closely related species *V. dahliae* mostly reproduces clonally with rare recombination events (Short et al. 2014). It lacks a saprophytic phase and is hard to control with fungicides, making resistance breeding and cultural practices the main management strategies. *B. cinerea*, causing gray mold, is a devastating necrotroph affecting numerous crops pre- and postharvest (Dean et al. 2012; Romanazzi and Feliziani 2014). It reproduces both sexually and asexually, and primarily spreads through airborne conidia. Despite being a generalist, population structure can reflect host specialization (Walker et al. 2015). Gray mold is traditionally managed with fungicides, but the emergence of resistance to all major classes of fungicides (FRAC, 2020), has prompted more integrated control approaches (Rotolo et al. 2018).

We hypothesize that in *F. culmorum* and *B. cinerea*, signatures of selection will be detectable in genes linked to fungicide resistance. Across all three species, we anticipate identifying evidence of adaptation related to pathogenicity. In predominantly clonal pathogens we expect adaptive changes to frequently span larger genomic regions rather than being confined to single genes. To ensure the robustness of our findings, we account for population structure throughout our analyses, allowing us to distinguish genuine signals of adaptation from background genetic variation.

## Results

### *Verticillium nonalfalfae* Shows Rare Sexual Reproduction and TE-associated Single Nucleotide Polymorphisms

**Fig. 1.**
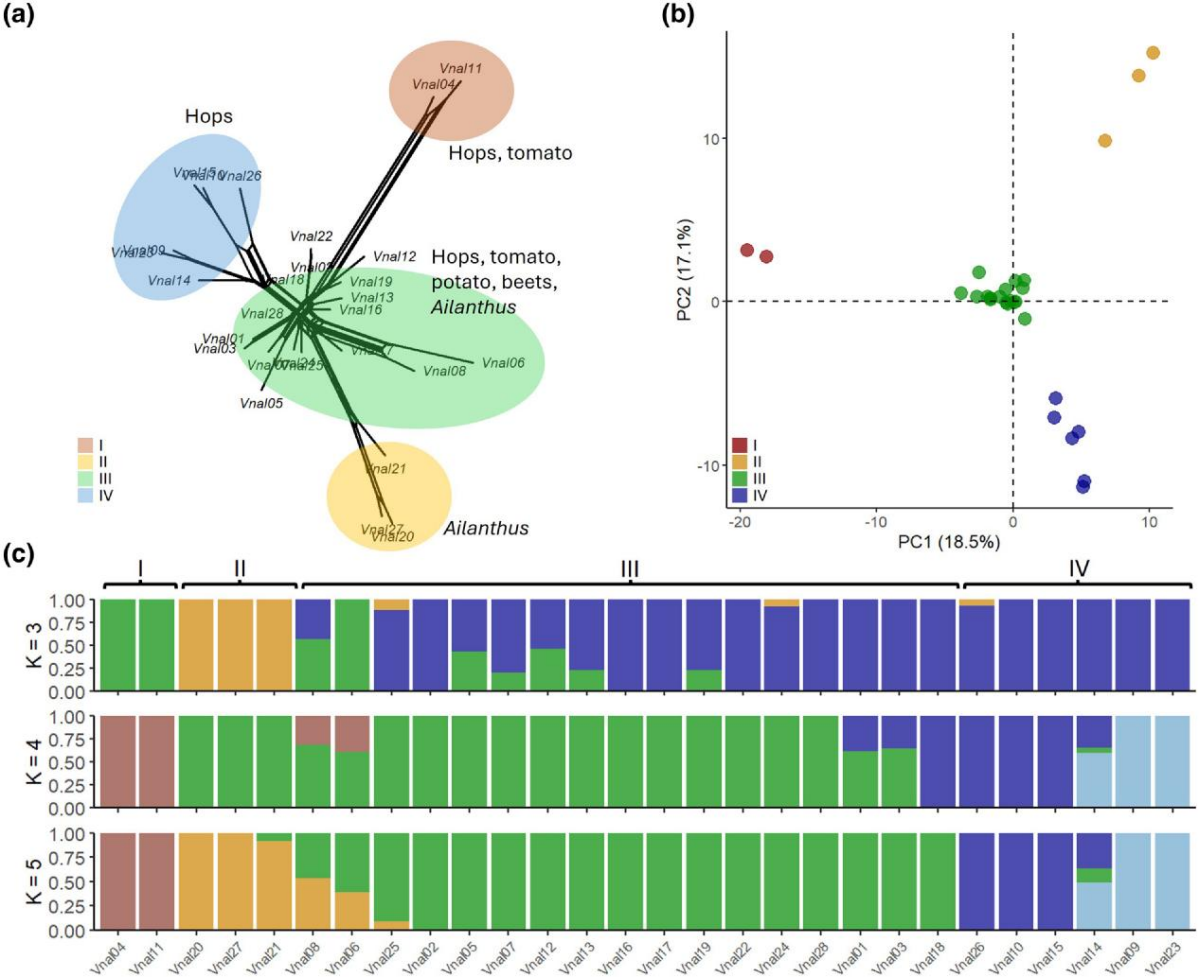
Population structure of *Verticillium nonalfalfae*. a) Network tree, b) PCA of the first two PCs explaining 35.2% of observed variation, c) admixture plots for *K* = 3, 4, and 5. Figures are based on 2,678 biallelic SNPs.

Single nucleotide polymorphisms (SNPs) calling across 38 *Verticillium* species (Table S1) yielded 9,804 high-quality biallelic SNPs after filtering. Phylogenetic analysis revealed that all 28 *V. nonalfalfae* isolates, collected between 1956 and 2016, formed a single low-diversity clade (Fig. S1). Within *V. nonalfalfae*, 2,678 high-quality biallelic SNPs were retained after filtering. Population structure analyses (network tree, Fig. 1a; PCA, Fig. 1b, Fig. S2a and b; STRUCTURE, Fig. 1c; clustering, Fig. S3) identified four clusters (I to IV), with group III being the largest (17 isolates, 61%). Group III contained isolates from a diverse range of hosts whereas groups II and IV consisted exclusively of isolates from *Ailanthus* and hops (*Humulus lupulus*), respectively. Strong clustering, low admixture, low within-group genetic diversity (mean *H* = 1.6; Table S2) and slow linkage disequilibrium (LD) decay (Fig. S4a) indicate predominant clonality, but nearly identical multilocus genotype counts in clone-corrected (21) and uncorrected (28) datasets, together with a low mean *r̅*d (0.05), suggest rare sexual reproduction (Fig. S5a–c).

**Fig. 2.**
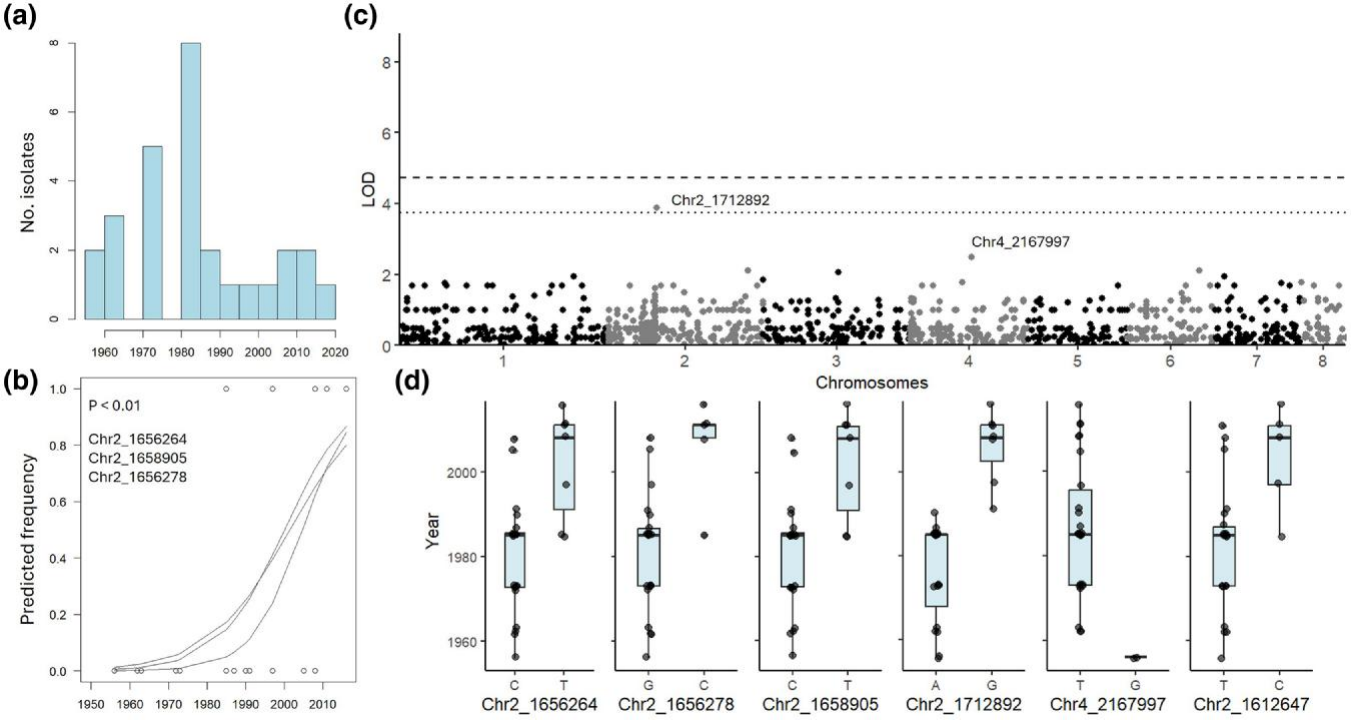
Temporal analysis of *Verticillium nonalfalfae*. a) Histogram of collection time points, b) binomial regression plot (*P* < 0.01), c) Manhattan plot of GWAS performed in R with first 10 PCs and vanRaden kinship as covariates, dashed line is Bonferroni threshold, dotted line is Bonferroni threshold—1, d) Boxplots showing allele change over time.

Annotation of reference genome Vnal01 identified 9,632 genes, a transposable element (TE) content of 5.1% (Table S1) and two *Starship* elements located on chromosome 2 and 3 (Table S3), respectively. Binomial regression identified two SNPs on chromosome 2 (chr2_1656264 and chr2_1658905, LOD = 2) that were associated with collection time (*P* < 0.001; Fig. 2a, Table 1, Table S4). Both are located within a 35 kb LTR-ERVL-MalR transposable element embedded in a 519 kb Arwing *Starship* element (Fig. S6, Table S3). The closest downstream (35 kb) annotated gene within the *Starship* element encodes a Zn2Cys6 fungal-type transcription factor (Table 1). GWAS identified an additional association 1 kb downstream of a phosphate transporter (chr2_1712892, LOD = 3.9; Fig. 2c) and <50 kb upstream of the *Starship* element. Another SNP was identified 0.6 kb downstream of a major facilitator superfamily (MFS) transporter on chromosome 4 (chr4_2167997, LOD = 2.7). Boxplots and regression plots (Fig. 2b and d) indicate that the temporal shift in SNP frequencies occurred around 1990.

**Table 1:** Significant SNPs and associated candidate genes

Species	Methods	Position	*P*	LOD	Gene	Uniprot/InterProScan	Dis (bp)
Vnal	Bino	2_1612647^a^	2.3E−02	1.64	Vnal01_002705^a^	NA	−43,596
Vnal	Bino	2_1656264^a,b^	9.9E−03	2.01	Vnal01_002706^a^	RING-type domain-containing protein	−40,953
Vnal	Bino	2_1656278^a,b^	1.4E−02	1.87	Vnal01_002707^a^	Zn2Cys6 fungal-type transcription factor	−35,344
Vnal	Bino	2_1656893^a,b^	1.6E−02	1.80	Vnal01_002708	DUF4440 domain-containing protein	+8,234
Vnal	Bino	2_1658905^a,b^	9.9E−03	2.01	Vnal01_002709	Enoyl reductase (ER) domain-containing protein	+10,358
Vnal	GWAS	2_1712892	1.2E−04	3.94	Vnal01_002722	Uncharacterized protein (Bacteria)	−4.430
Vnal	…	…	…	…	Vnal01_002723	Phosphate transporter	+1.310
Vnal	GWAS	4_2167997	1.9E−03	2.71	Vnal01_005957	Aflatoxin biosynthesis ketoreductase nor-1	−558
Vnal	…	…	…	…	Vnal01_005958	Major facilitator superfamily (MFS), Multidrug resistance protein D	+678
Fcul	Bino	1_541701	7.7E−06	5.12	Fcul02_000150	Egh16-like virulence factor	−284
Fcul	GWAS	1_541701	1.7E−05	4.76	Fcul02_000151	Peptide hydrolase (Zn)	+850
Fcul	Bino	1_3034682	2.6E−05	4.58	Fcul02_000949	Peptidyl-prolyl cis-trans isomerase (CYP1)	−406
…	…	…	…	…	Fcul02_000950	Intramembrane protease 2	+71
Fcul	GWAS	2_7145691	1.7E−06	5.77	Fcul02_005748	Heterokaryon incompatibility domain-containing protein	−1,325
Fcul	…	…	…	…	Fcul02_005749	High nicotine affinity transporter, MFS	+542
Fcul	GWAS	3_7827009	1.1E−05	4.96	Fcul02_008570	Pantothenate transporter liz1, MFS	−829
…	…	…	…	…	Fcul02_008571	Uncharacterized protein	+371
Fcul	GWAS	4_11090998	1.5E−05	4.82	Fcul02_012256	RRM domain-containing protein	−1,361
Fcul	…	…	…	…	Fcul02_012257	HNH nuclease domain-containing protein	+1,833
Fcul	Bino	4_11141387	2.7E−05	4.57	Fcul02_012267	Uncharacterized protein	−1,769
Fcul	…	…	…	…	Fcul02_012268	Zn2Cys6 fungal-type domain-containing protein, Mg2+ transporter protein, CorA-like/Zinc transport protein ZntB	+853
Bcin	GWAS	1_2936826	2.4E−07	6.61	Bcin04_000811	Argininosuccinate synthase	−491
Bcin	GWAS	5_2616829	2.0E−06	5.71	Bcin04_003972	PX domain-containing protein	0
Bcin	GWAS	6_1565647	1.0E−10	10.00	Bcin04_005341	Zn2Cys6 fungal-type transcription factor	0
Bcin	GWAS	9_196846	9.7E−07	6.01	Bcin04_005702	Kinesin	−2,364
Bcin	…	…	…	…	Bcin04_005703	CCHC-type Zinc Finger Nucleic Acid Binding	+1,131
Bcin	GWAS	10_1674774	1.2E−06	5.93	Bcin04_007601	Carnosine N-methyltransferase	0
Bcin	Bino	10_1807139	1.1E−03	2.96	Bcin04_007635	MFS	−15
Bcin	…	…	…	…	Bcin04_007636	Short-chain dehydrogenase/reductase ABA4	+675

Vnal = *Verticillium nonalfalfae,* Fcul = *Fusarium culmorum*, Bcin = *Botrytis cinerea*. Methods indicate if SNP was found with GWAS or binomial regression (Bino). Position is listed as chr_position. Gene is annotation of the reference genome; corresponding protein sequences are found in Table S4. Uniprot column states the predicted functions by Uniprot. Distance in base pairs down (−) or upstream (+) from SNP.

^a^Within Vnal *Arwing Starship* (Chr2: 1144581 → 1663655).

^b^Within LTR_RRVL-MalR TE (Chr2: 1,625,269 → 1,660,599).

### *Fusarium culmorum* Shows Frequent Recombination and Gene-associated SNPs

**Fig. 3.**
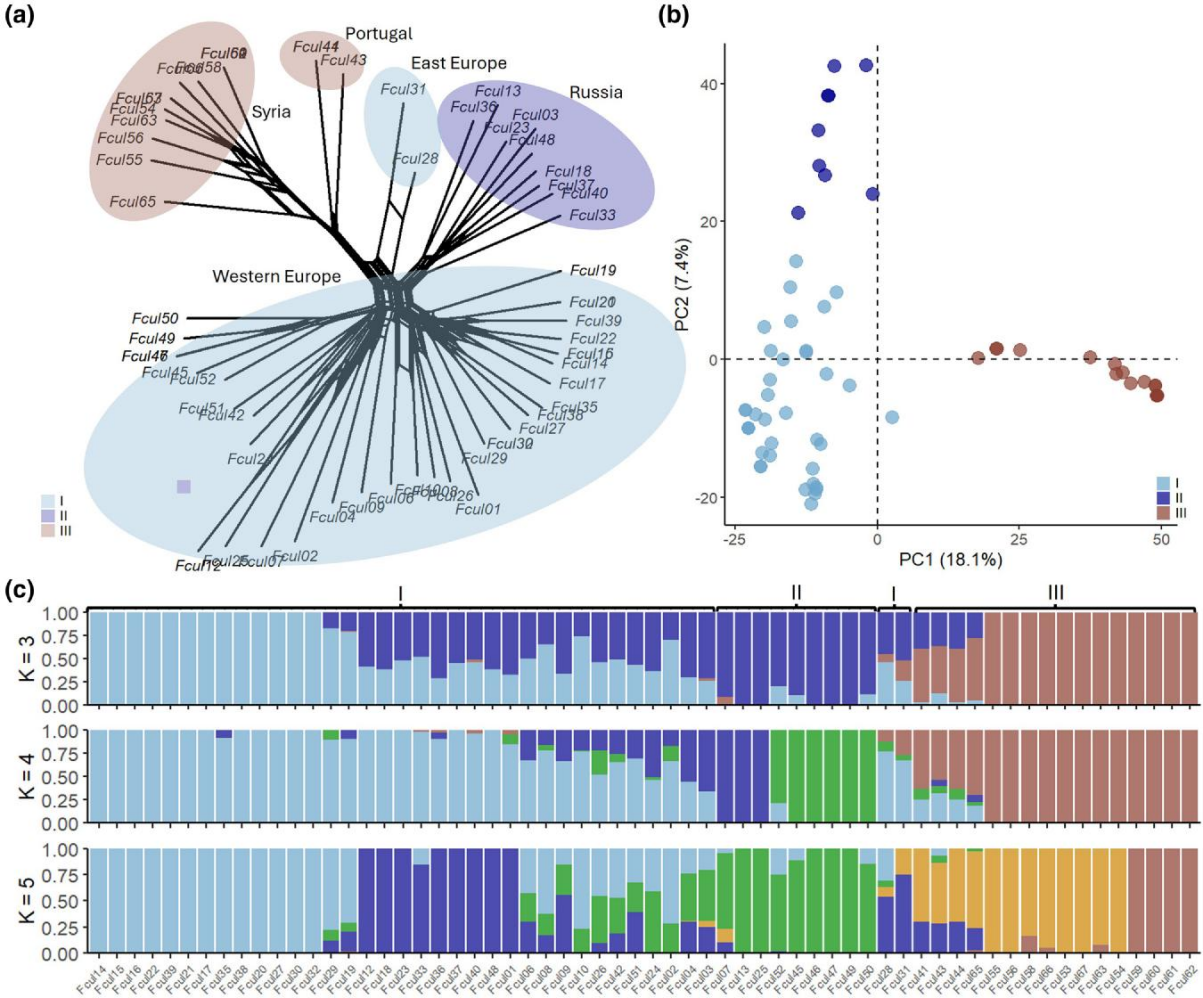
Population structure of *Fusarium culmorum*. a) Network tree, b) PCA of the first two PCs explaining 25.5% of observed variation, c) admixture plots for *K* = 3, 4, and 5. Figures are based on 22,596 biallelic SNPs.

SNP calling across 67 *Fusarium* species (Table S1) yielded 9,804 high-quality biallelic SNPs after filtering. Phylogenetic analysis placed all 62 *F. culmorum* isolates into a single clade (Fig. S7). Within *F. culmorum*, high-quality biallelic 22,596 SNPs were retained after filtering. Population structure analyses (network tree, Fig. 3a; PCA, Fig. 3b, Fig. S2c and d; STRUCTURE, Fig. 3c; clustering, Fig. S8) identified three partially overlapping clusters with notable admixture. High within-group diversity (*H* = 2.9; Table S2), rapid LD decay (Fig. S4c), and low *r̅*d (0.025; Fig. S5e and f) all indicate frequent sexual reproduction. The highest diversity was observed in the Euro-Mediterranean region, which overlaps with one of the primary centers of wheat domestication and could represent a center of diversity of *F. culmorum*.

**Fig. 4.**
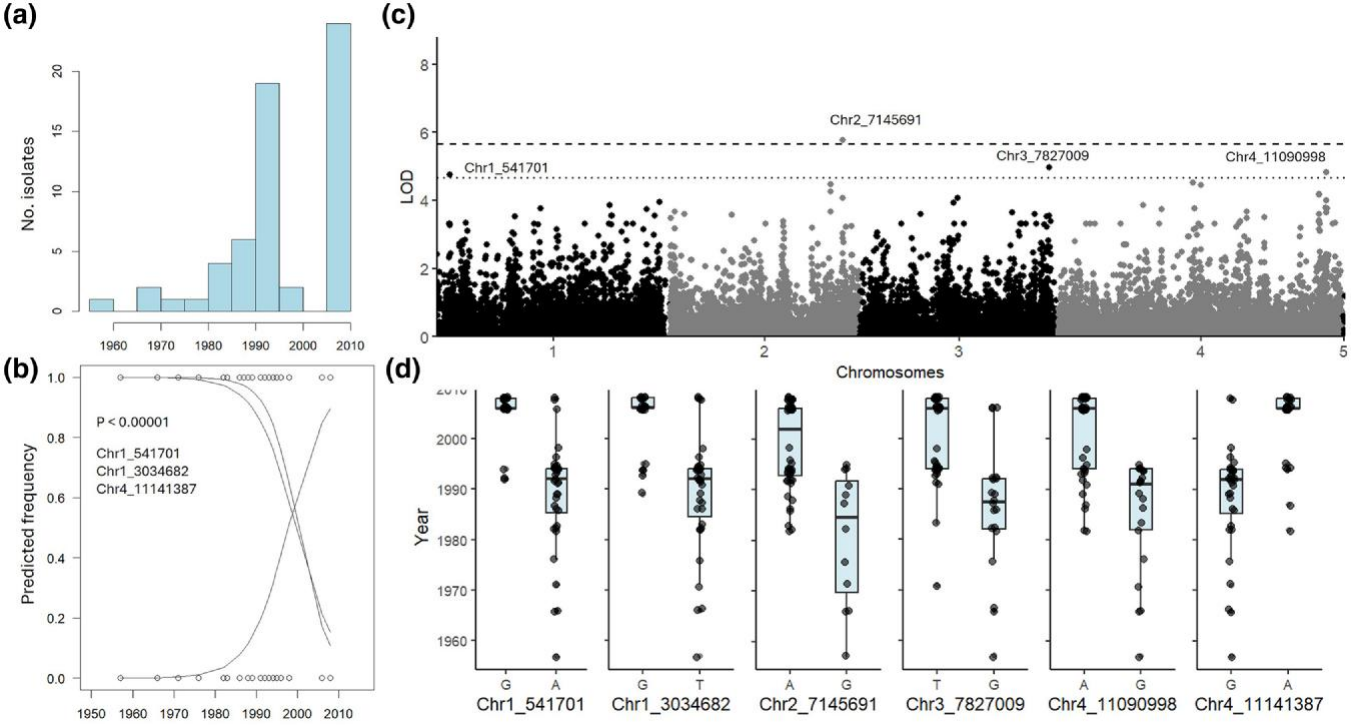
Temporal analysis of *Fusarium culmorum*. a) Histogram of collection time points, b) binomial regression plot (*P* < 1E–05), c) Manhattan plot of GWAS performed in R with first 10 PCs and vanRaden kinship as covariates, dashed line is Bonferroni threshold, dotted line is Bonferroni threshold—1, d) Boxplots showing allele change over time.

Annotation of reference genome Fcul02 identified 12,303 genes, a TE content of 4.3% (Table S1) and two *Starship* elements located on chromosome 1 and 2, respectively (Table S3). Significant temporal associations were identified with three SNPs (chr1_541701, chr1_3034682, chr4_11141387; LOD < 4.6; Fig. 4b, Table 1, Table S4). These SNPs are located immediately adjacent to coding genes (<0.85 kb), including a Zn-dependent peptidase, an Egh16-like virulence factor, a CYP1 protein, and an intramembrane protease. GWAS confirmed and extended these associations (Fig. 4c and d), identifying additional significant SNPs near a Zn2Cys6 transcription factor (chr4_11141387, LOD = 4.6, 0.85 kb) and a MFS transporter (chr2_7145691, LOD = 5.8, 0.5 kb). Other associated SNPs were linked to heterokaryon incompatibility proteins and transporters. Temporal shifts in allele frequencies were dated between 1990 and 2000 (Fig. 4b and d).

### *Botrytis cinerea* Shows High Diversity and Gene-associated SNPs

**Fig. 5.**
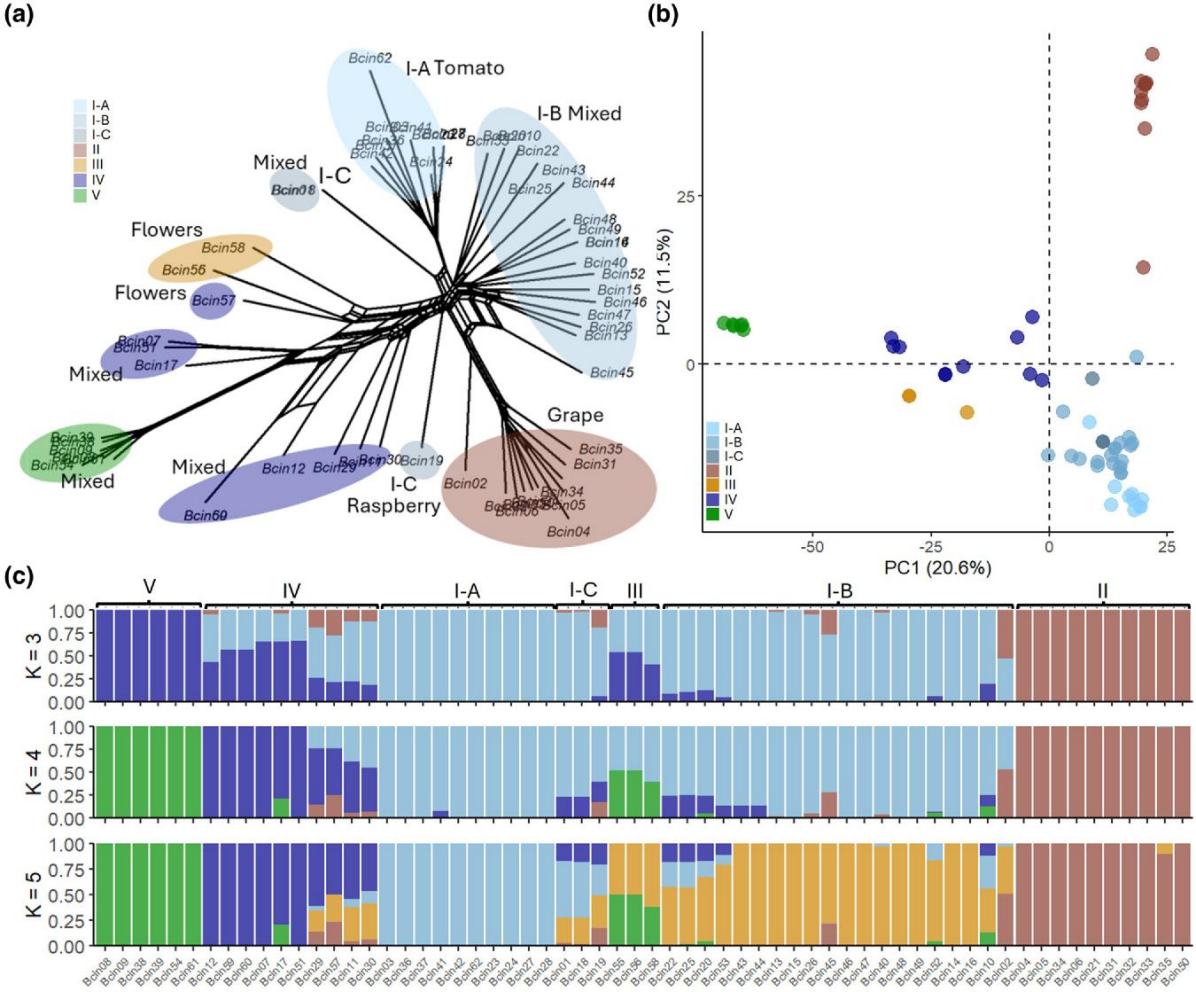
Population structure of *Botrytis cinerea.* a) Network tree, b) PCA of the first two PCs explaining 32.1% of observed variation, c) admixture plots for *K* = 3, 4, and 5. Figures are based on 22,784 biallelic SNPs.

SNP calling across 69 *Botrytis* isolates (plus *Sclerotinia sclerotiorum* as outgroup) yielded 17,655 high-quality variants. Phylogenetic analysis confirmed all 62 *B. cinerea* isolates belong to a single clade, though with considerable intraspecific diversity (Fig. S9). Within *B. cinerea*, high-quality biallelic 22,784 SNPs were retained after filtering. Although 16 core chromosomes have been described for *B. cinerea* (Van Kan et al. 2017), successful mapping was only achieved for 10 (Fig. S10), indicating either intraspecific chromosomal variation or that *B. cinerea* is a species complex. Population structure analyses (network tree, Fig. 5a; PCA, Fig. 5b, Fig. S2e and f; STRUCTURE, Fig. 5c; clustering, Fig. S11) identified five genetic clusters (I to V), including subgrouping within cluster I (A to C). Subgroup I-A was exclusively associated with tomato and all isolates in group II were collected from grape. High diversity (*H* = 2.2; Table S2), rapid LD decay (Fig. S4e), and nearly identical multilocus genotype counts in clone-corrected (54) and uncorrected (55) datasets (r̅d = 0.05; Fig. 5g–i) all indicate frequent recombination. Notably, cluster V shows no admixture and may represent a cryptic lineage within the *B. cinerea* complex.

**Fig. 6.**
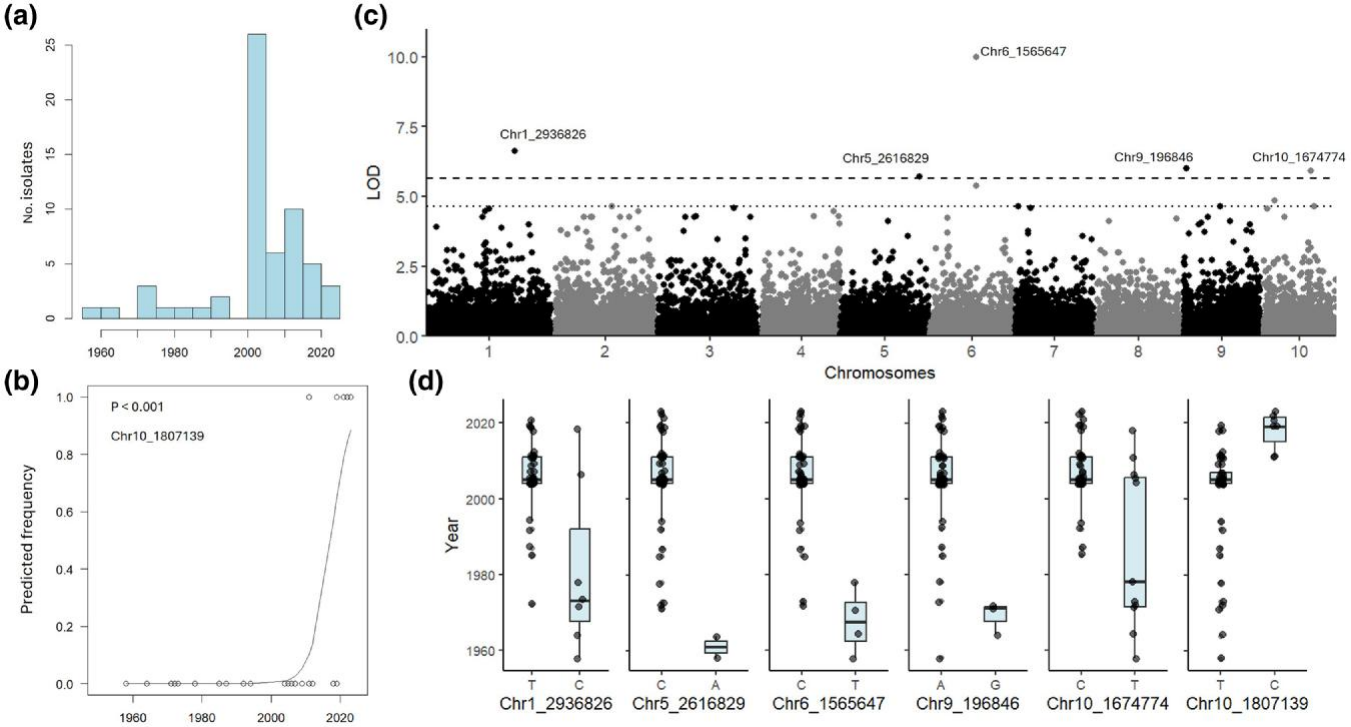
Temporal analysis of *Botrytis cinerea*. a) Histogram of collection time points, b) binomial regression plot (*P* < 0.001), c) Manhattan plot of GWAS performed in R with first 10 PCs and vanRaden kinship as covariates, dashed line is Bonferroni threshold, dotted line is Bonferroni threshold—1, d) Boxplots showing allele change over time.

Annotation of reference genome Bcin04 identified 12,159 genes, a TE content of 10.6% (Table S1) and one *Starship* elements located on chromosome 1 (Table S3). Binomial regression identified a significant SNP on chromosome 10 (chr10_1807139, LOD = 3; Fig. 6b, Table 1, Table S4), located 15 bp upstream of an MFS transporter and 0.7 kb downstream of a short-chain dehydrogenase/reductase. GWAS further identified five associated SNPs (Fig. 6c and d), including one within a Zn2Cys6 fungal-type transcription factor (chr6_1565647, LOD = 10), and one near a kinesin and a CCHC-type zinc finger nucleic acid-binding protein (chr9_196846, LOD = 6, 1.1 kb). Temporal shifts in SNP frequencies varied, with changes in chromosomes 1, 5, 6, and 9 occurring between 1960 and 1980, and the chr10_1807139 SNP shifting more recently around 2010 (Fig. 6b and d).

## Discussion

This study used historical collections and publicly available genomes of three fungal crop pathogens to investigate their population structure and genetic changes over time. The results reveal contrasting patterns of sexual reproduction and genomic diversification across the species, while highlighting shared functional pathways underlying adaptation. In the predominantly clonal *V. nonalfalfae*, identified SNPs are embedded within or close to TEs and *Starship* elements, whereas in the two sexual pathogens *F. culmorum* and *B. cinerea* they consistently map within or adjacent to coding genes. Despite this contrast, all three species show SNPs repeatedly close to or within MFS transporters and Zn2Cys6 fungal-type transcription factors (Table 1), underscoring their potential roles in fungal adaptation.

Both *B. cinerea* and *F. culmorum* displayed clear signatures of sexual recombination, including high within-group genetic diversity, rapid LD decay, and widespread admixture. Although sexual reproduction in *B. cinerea* has long been recognized (Faretra et al. 1988), *F. culmorum* was previously thought to be asexual due to the absence of a teleomorph (Scherm et al. 2013). Our results, consistent with earlier microsatellite studies (Miedaner et al. 2013), provide strong evidence for frequent recombination in this species. In *B. cinerea*, frequent recombination coexists with strong genetic clustering and limited admixture between groups, suggesting barriers to gene flow. Moreover, only 10 of the described 16 “core” chromosomes (Van Kan et al. 2017) could be consistently mapped across isolates, supporting the idea that *B. cinerea* may not represent a single cohesive species but rather a species complex (Saito et al. 2016). In contrast to *B. cinerea* and *F. culmorum*, *V. nonalfalfae* showed strong clustering, low diversity, and slow LD decay, consistent with primarily clonal reproduction. However, occasional recombination appears to occur, as also observed in in related *V. dahliae* (Short et al. 2014).

The central finding of this study is the clear difference in the genomic locations of SNPs associated with temporal change between clonal and sexual fungi. In *V. nonalfalfae*, identified SNPs were embedded within or near TEs and a *Starship* element, consistent with TE-mediated diversification in clonally reproducing fungi (Dong et al. 2015; Torres et al. 2020). Similar reliance on TE and *Starship* activity has been documented in related Verticillium species (Sato et al. 2025), and predominantly asexual *Colletotrichum lupini* (Alkemade et al. 2024), *F. oxysporum* (Peck et al. 2024; López Díaz et al. 2025). and *Macrophomina phaseolina* (Gluck-Thaler et al. 2022). Moreover, in the predominantly asexual *Monilinia* species, DMI fungicide resistance was shown to be TE-driven (Durak and Özkılınç 2025). In contrast, in the recombining pathogens *F. culmorum* and *B. cinerea*, identified SNPs were consistently located within or adjacent to protein-coding genes, as was also seen for sexual *Blumeria graminis* (Jigisha et al. 2025) and *Z. tritici* (Feurtey et al. 2023). This suggests a divergence in mechanisms of adaptive evolution where clonal fungi may depend on structural variation and TE activity, and sexual fungi accumulate adaptive point mutations directly in or near coding genes, but this should be tested for more species to confirm.

Despite differences in lifestyle and genomic architecture, all three pathogens showed significant associations close to or within genes encoding for Zn2Cys6 fungal-type transcription factors and MFS transporters. Zn2Cys6 proteins are fungal-specific regulators implicated in stress responses, secondary metabolism, and virulence (Tianqiao et al. 2021; Yin et al. 2021; John et al. 2024). In *B. cinerea*, they control reproduction and virulence (Lu et al. 2023), while in *F. culmorum* they were shown to regulate growth and pathogenicity (Yang et al. 2024). Their repeated association across pathogens in this study suggests a central role in long-term adaptation to hosts and environments.

MFS transporters are similarly recurrent, with functions in efflux-mediated detoxification, nutrient acquisition, and virulence (Wu et al. 2016; Lin et al. 2018). In *B. cinerea*, MFS transporters confer fungicide tolerance and are required for pathogenicity (Vela-Corcía et al. 2019). In *F. culmorum*, we identified SNPs linked to a nicotine-affinity transporter and a pantothenate transporter, both of which may contribute to detoxification and pathogenicity (Kamau et al. 2025; Wang et al. 2025). The association with MFS transporters for all three fungal species indicate there might be links to fungicide resistance, but resistance through detoxification is not commonly reported in fungal pathogens (Lucas et al. 2015).

Additional loci in *F. culmorum* included a CYP1 gene, previously implicated in virulence and as a drug target in other fungi (Viaud et al. 2002; Chen et al. 2011), and an Egh16-like effector, conserved across plant-pathogenic fungi (Xue et al. 2002; Moonjely and Trail 2025). In *V. nonalfalfae*, the strongest temporal association was near a phosphate transporter gene, consistent with roles in nutrient acquisition and virulence (Deng et al. 2015; Bhalla et al. 2022). Together, these findings reinforce the importance of both metabolic adaptation and effector-mediated pathogenicity.

Interestingly, none of the identified SNPs were located in common fungicide target genes such as β-tubulin, cytochrome b, CYP51, or succinate dehydrogenase subunits, which frequently carry resistance mutations in other fungal pathogens (Hawkins and Fraaije 2021; Dorigan et al. 2023; Yin et al. 2023; Oliver et al. 2024). This aligns with recent findings of purifying selection and low variability in fungicide target loci of *F. culmorum* and *V. nonalfalfae* (Wong et al., Submitted) but contrasts with a population studies on *B. graminis* (Jigisha et al. 2025; Minadakis et al. 2025), *Aspergillus fumigatus* (Snelders et al. 2025), and *Z. tritici* (Feurtey et al. 2023). Instead, our results point toward alternative mechanisms of adaptation, particularly transport-mediated detoxification and transcriptional regulation.

Our results also provide some new insights into population structure of these pathogens. *V. nonalfalfae*, generally considered a broad host range pathogen (Fradin and Thomma 2006), showed signs of partial host specialization. Genetic groups II and IV were exclusively associated with *Ailanthus* and hops, respectively. Earlier host range testing with isolate Vnal28 (VnAa140), clustering within group III, revealed specificity to *A. altissima* (Kasson et al. 2015), though testing on other common hosts such as tomato or hops was not performed. Host specialization within broad host range species has also been reported in the closely related *V. dahliae* and *V. longisporum* (Zeise and Von Tiedemann 2002), as well as in *B. cinerea* (Mercier et al. 2021). Consistent with Mercier et al. (2021), this study confirmed host associations in *B. cinerea*, showing genetic groups specific to grapevine and tomato.

In *F. culmorum*, population structure was strongly linked to geography. Isolates from Australasia and the Americas clustered with Western European isolates, suggesting a Western European source population. The observed separation between Western and Eastern European populations, and the close relatedness of Portuguese and Syrian isolates, parallels global wheat genetic diversity patterns (Balfourier et al. 2019). All genetic diversity detected was contained within the Euro-Mediterranean region, which overlaps with centers of cereal domestication, supporting this region as the likely center of diversity for *F. culmorum*. Future work should test whether isolates from noncereal hosts, such as leek (Koike et al. 2003), represent additional diversity.

This study demonstrates the potential of temporal association analysis in historical collections for uncovering the evolutionary dynamics of fungal pathogens. By treating time as a trait and integrating it into genome-wide association frameworks, we were able to identify candidate loci without requiring additional phenotyping. Importantly, the contrast between TE-associated SNPs in clonal pathogens and gene-associated SNPs in recombining pathogens provides a novel perspective on how reproductive mode shapes adaptive trajectories. Limitations remain, including the scarcity of older isolates and uneven sampling, but the approach holds promise. While the findings presented here remain largely exploratory, they establish a foundation for the field of historical genomics in fungi. Expanding this approach to a wider range of species, particularly by leveraging the wealth of preserved isolates stored in global fungal collections, could unlock significant evolutionary insights. These collections represent a largely untapped resource and can serve as a genomic treasure trove with the potential to deepen our understanding of pathogen adaptation over time.

## Materials and Methods

### Strain and Data Collection

For each species, all publicly available genomes were used at the time of download (6-2025). For *V. nonalfalfae* 15 strains were obtained from CABI and 12 genomes were publicly available (Table S1, Wong et al. Submitted; Kasson et al. 2019; Berger et al. 2020; Seidl et al. 2020). These strains were collected between 1956 and 2016 from Europe, North America, and Australia. Genomes of *V. dahliae*, *V. alfalfae*, *V. albo-atrum*, *V. longisporum*, and *V. nubilium* were used as outgroup (Klosterman et al. 2011; Faino et al. 2015; Shi-Kunne et al. 2018; Seidl et al. 2020). For *F. culmorum* six strains were obtained from CABI and 56 genomes were publicly available (Miedaner et al. 2021; Turco et al. 2021; Kulik et al. 2022; Jeong et al. 2024; Wong et al., Submitted). These *F. culmorum* strains were collected between 1957 and 2008, from Europe, Syria, Russia, China, the USA, and Australia, and were primarily associated with cereal crops, most notably wheat (75%; Table S1). Genomes of *F. graminearum*, *F. asiaticum*, *F. pseudograminearum*, and *F. cortaderiae* were used as outgroups (Cuomo et al. 2007; Gardiner et al. 2012; Jeong et al. 2024). For *B. cinerea*, seven strains were obtained from CABI and 55 were publicly available (Blanco-Ulate et al. 2013; Van Kan et al. 2017; Atwell et al. 2018; Mercier et al. 2021; Patricio-Hernández et al. 2023; Adhikari et al. 2025). These strains were collected between 1958 and 2023 from Europe and North America and were primarily associated with fruit crops including grape, tomato, and berries (Table S1). Genomes of *B. aclada*, *B. byssoidea*, *B. calthae*, *B. porri*, *B. sinoalli*, and *S. sclerotiorum* were used as outgroups (Amselem et al. 2011; Coca-Ruiz et al. 2024). Genome data were collected as raw reads (SRA) when available or as assemblies. Collection times spanned from 1953 to 2023 (Table S1), which covers the main period of chemical intensification of agriculture and development of single-site fungicides.

### DNA Extraction, Sequencing, and Quality Control

DNA extraction and sequencing were performed for *B. cinerea* isolates Bcin07-12 and *V. nonalfalfae* isolates Vnal03, Vnal06, and Vnal07 (Table S1). Cryopreserved fungal spores were rehydrated with distilled water for 30 min and spread on potato dextrose agar (PDA) at 22 °C. Cultures were incubated at 21 °C for 7 d then mycelium was harvested, snap frozen using liquid nitrogen and ground with 2 mm steel beads using a TissueLyser II (QIAGEN, Germany). DNA was extracted with a DNeasy Plant Mini Kit (QIAGEN, Germany) following the manufacturer's instructions, with 30 min lysis buffer incubation. Purified extracted DNA was sent to Oxford Genomics Centre (Oxford, UK) for sequencing (paired-end 150 bp) on an Illumina NovaSeq 6000 platform. Raw reads were trimmed for adapter sequences and filtered for a phred quality of 20 and length of 50 bp using FastP v. 0.23.4 (Chen et al. 2018). Quality was evaluated using FastQC v. 0.12.1 (Andrews 2010).

### Phylogenetics

SNPs were called by mapping reads to reference genomes Bcin04, Fcul02, or Vnal01, respectively (Table S1), using BWA v. 0.7.17-r1188 (Li, 2013). Resulting SAM files were converted to BAM files and indexed and sorted using SAMtools v. 1.17 (Danecek et al. 2021). Variant calling was performed using mpileup of BCFtools v. 1.14 (Danecek et al. 2021) and variants were filtered for quality (Q20), minimum sequencing depth (2), mean sequencing depth (5), minor allele count (2), minor allele frequency (0.01), and missing data (0.90) using VCFtools v. 0.1.16 (Auton and Marcketta 2009). Additional pruning with a sliding window of 1 kb was performed to limit number of SNPs when necessary. Phylogenetic analysis was performed with IQ-TREE2 (-m GTR+ASC -B 1000 -alrt 1000; Minh et al. 2020), and ape v5.7-1 in R (Paradis and Schliep 2019) and visualized using Itol (Letunic and Bork 2007). Network trees were constructed using Phangorn in R (Schliep 2011).

### Population Genetics

Population genetic analyses were performed separately for each species following phylogenetic verification. Prior to filtering and pruning, all outgroup strains were excluded to ensure within-species analyses. Genetic clustering within populations often represents a continuum rather than sharply defined groups and as no single method alone provides a complete picture we integrated multiple complementary approaches. Population structure was assessed via principal component analysis (PCA) of SNP variation using the *glPca* function and Discriminant Analysis of Principal Components (DAPC) using *dapc*, both from *adegenet* v2.1.10 (Jombart 2008). The number of principal components and clusters to retain was optimized using xvalDAPC. Admixture and clustering patterns were further evaluated using ADMIXTURE v1.3.0 (Alexander et al. 2009) with *K* values from 1 to 10 under default settings and fitting BIC and AIC models.

Genetic variation within and among populations was quantified using AMOVA (Excoffier et al. 1992) with 1,000 permutations, performed on both clone-corrected and noncorrected datasets with the amova function in *poppr* (Kamvar et al. 2014). Genetic differentiation (*F*_st_) was estimated using the genet.dist function in *hierfstat* (Goudet 2005). Multilocus genotypes (MLGs) were identified using the mlg.filter function in *poppr*, with a threshold determined via the cutoff predictor based on Euclidean distance. Clone correction was applied using a threshold of 0.05. Diversity statistics and minimum spanning networks (MSNs) were generated for both clone-corrected and noncorrected datasets using *poppr* and poppr.msn.

Multilocus linkage disequilibrium was assessed using the index of association (IA) and its standardized form (*r̅*d), both calculated with 999 permutations via the ia function in *poppr*. Values of (*r̅*d), approaching 0 suggest random mating, while those near 1 indicate clonality. Linkage decay was estimated using PLINK v1.9 (Chang et al. 2015) with a 1,000 bp sliding window. Average linkage disequilibrium was computed using the Python script ld_decay_calc.py from speciationgenomics/scripts.

### Temporal Association

Associations over time were performed through whole genome association (GWAS) analysis, using a mixed linear model (MLM) using statgenGWAS (van Rossum et al. 2020) in R following the method described in (Kang et al. 2010). The first 10 principal components (PCs) were included as covariates to control for population structure, and a vanRaden kinship matrix was included to account for cryptic relatedness (VanRaden 2008). An efficient mixed model association (EMMA) algorithm was used to estimate the variance components (Kang et al. 2008). A Bonferroni corrected LOD threshold was used ($-log10\left(\frac{0.05\;}{n\;SNPs}\right)$) to identify significant SNPs. Manhattan and quantile–quantile (Q–Q) plots were generated within *statgenGWAS*. We also ran binominal regression for each SNP to asses changes in allele frequency over time. Only samples with nonmissing genotype calls at a given site were included (ie excluding indels). The following model was fitted: $\left(log\left(\frac{\;p_{ij}\;}{1\;-\;p_{ij}}\right)=\;\beta_{i}^{0}\;+\;\beta_{i}^{1}\;\cdot \;x_{j}\right)$, here *pᵢⱼ* is the probability that strain *j* carries the alternate allele at SNP *i*, and *x_j_* is the year of isolation for strain *j*. From each fitted model, the slope coefficient *β₁ᵢ* and its associated *P*-value were extracted to assess whether the frequency of the alternate allele showed a statistically significant (*P* < 0.01) temporal trend. Closest genes within a 10 kb range up- and downstream or on the same TE were considered as candidate genes.

### Genome Annotation

Gene annotation of reference genomes (Table S1) was performed on masked genomes with Funannotate v1.8.17 (Palmer and Stajich 2020) using publicly available transcripts and proteins of closely related species (Table S1) and pretrained versions (fusarium_graminearum, verticillium_longisporum1, and botrytis_cinerea) of Augustus v3.5.0 (Keller et al. 2011). Possible functions were predicted using BLASTP, Uniprot and InterProScan v5.63. Reference genome specific repetitive elements were identified using Repeatmodeler v2.0.4 (Flynn et al. 2020) with options *-engine ncbi* and *-LTRStruct*. Two consensus (identity and coverage > 80%) TE libraries were created, one from the Sordariomycetes Dfam database (Wheeler et al. 2012) for *V. nonalfalfae* and *F. culmorum*, and one from the Leotiomycetes database for *B. cinerea*. Repeats were classified with RepeatClassifier. Complex repeats were annotated using two successive rounds of RepeatMasker (cutoff = 250), with the first round being performed with the appropriate Dfam consensus library and the second round with each species-specific repeat library. Giant transposable elements (*Starship*s) were annotated using Starfish v0.3.3 (Gluck-Thaler and Vogan 2024) with default parameters. Candidate *Starship*s were identified based on sequence similarity to curated reference elements and the presence of hallmark tyrosine recombinase (YR) “captain” genes, using BLAST v2.12.0+ (Camacho et al. 2009). To reduce false positives, predicted elements were further validated through synteny analysis with closely related species using nucmer from MUMmer v4.0.0rc1 (Marçais et al. 2018), requiring alignments of ≥10 kb with >80% nucleotide identity.

## Supplementary Material

evaf241_Supplementary_Data

## Data Availability

All genomic data used in this study is publicly available on https://www.ncbi.nlm.nih.gov/. See Table S1 for genome accession codes. Used annotations and VCF files are publicly available at doi: 10.5281/zenodo.17867089.
