# Supplementary material for: Genomic Insights into Historical Adaptation of Three Key Fungal Plant Pathogens
Source: Genome Biol Evol. 2025 Dec 11;17(12):evaf241. doi: 10.1093/gbe/evaf241 (PMC12720139; doi:10.1093/gbe/evaf241)
Supplement: evaf241_Supplementary_Data [file evaf241_supplementary_data.zip › Hist_genomics_paper_GBE_Supp_Figures.pdf]

## Genomic insights into historical adaptation of three key fungal plant pathogens

Joris A. Alkemade[1,2*], Edgar L. Y. Wong[1,3], Alan G. Buddie[4], Matthew J. Ryan[4], Timothy G. Barraclough[1,2]

1: Department of Biology, University of Oxford, Oxford, UK
2: Calleva Centre, Magdalen College, Oxford, UK
3: Senckenberg Biodiversity and Climate Research Centre, Frankfurt am Main, Germany
4: CAB International (CABI), Ascot, UK

*Corresponding author: joris.alkemade@magd.ox.ac.uk

### Supplementary figures

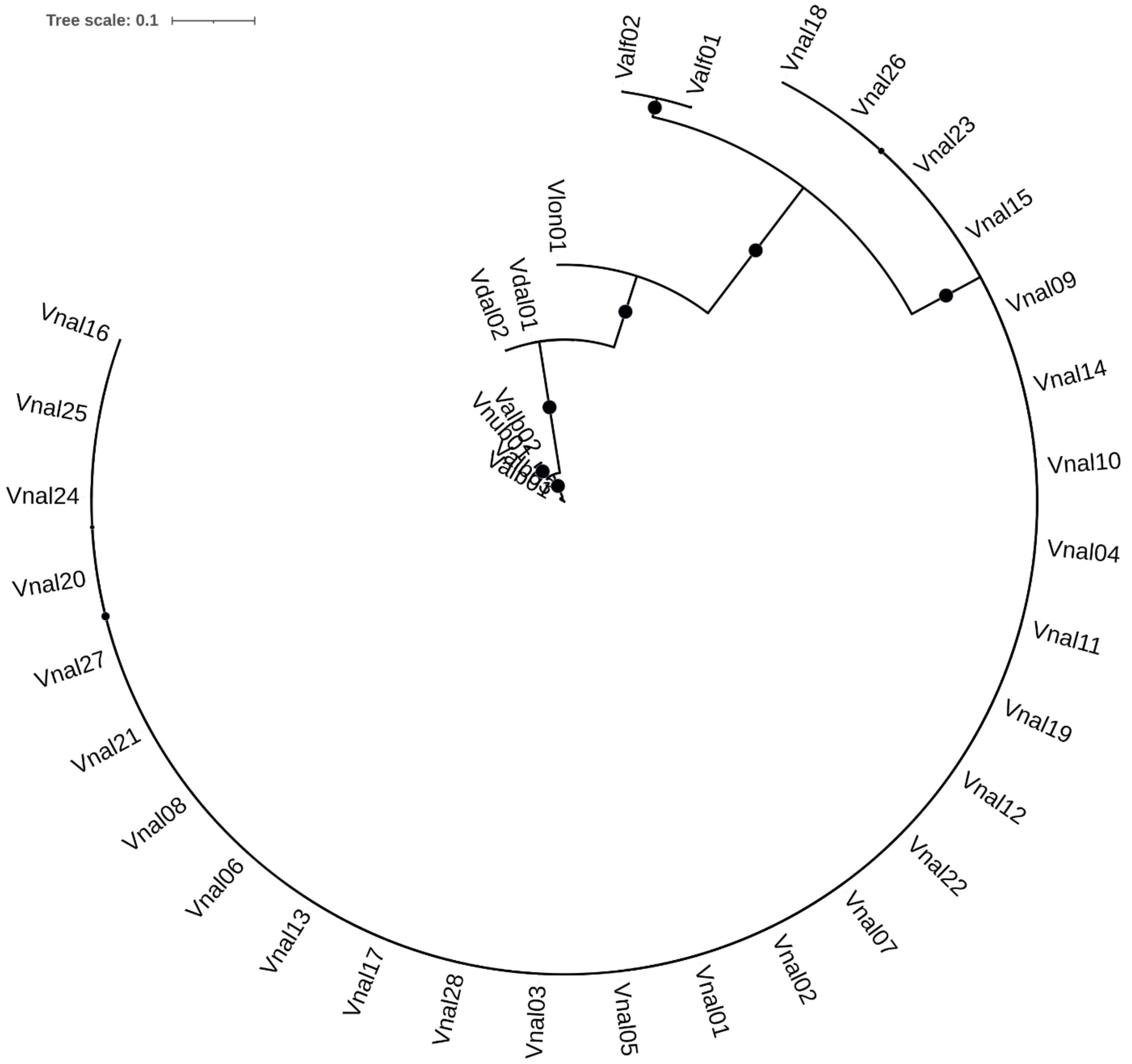


**Figure S1: *Verticillium nonalfalfae* phylogeny.** Maximum likelihood tree from 9,804 SNPs of 37 *Verticillium* isolates. Bootstrap support values (>90) are indicated with a black dot at each node. Tree is rooted to Valb01. Valb = *V. albo-atrum,* Vnub = *V. nubilum*, Vdal = *V. dahliae*, Valf = *V. alfalfae* and Vnal = *V. nonalfalfae*. Valb02 is *F. nubilium.* Vnal01 was used as the reference genome.

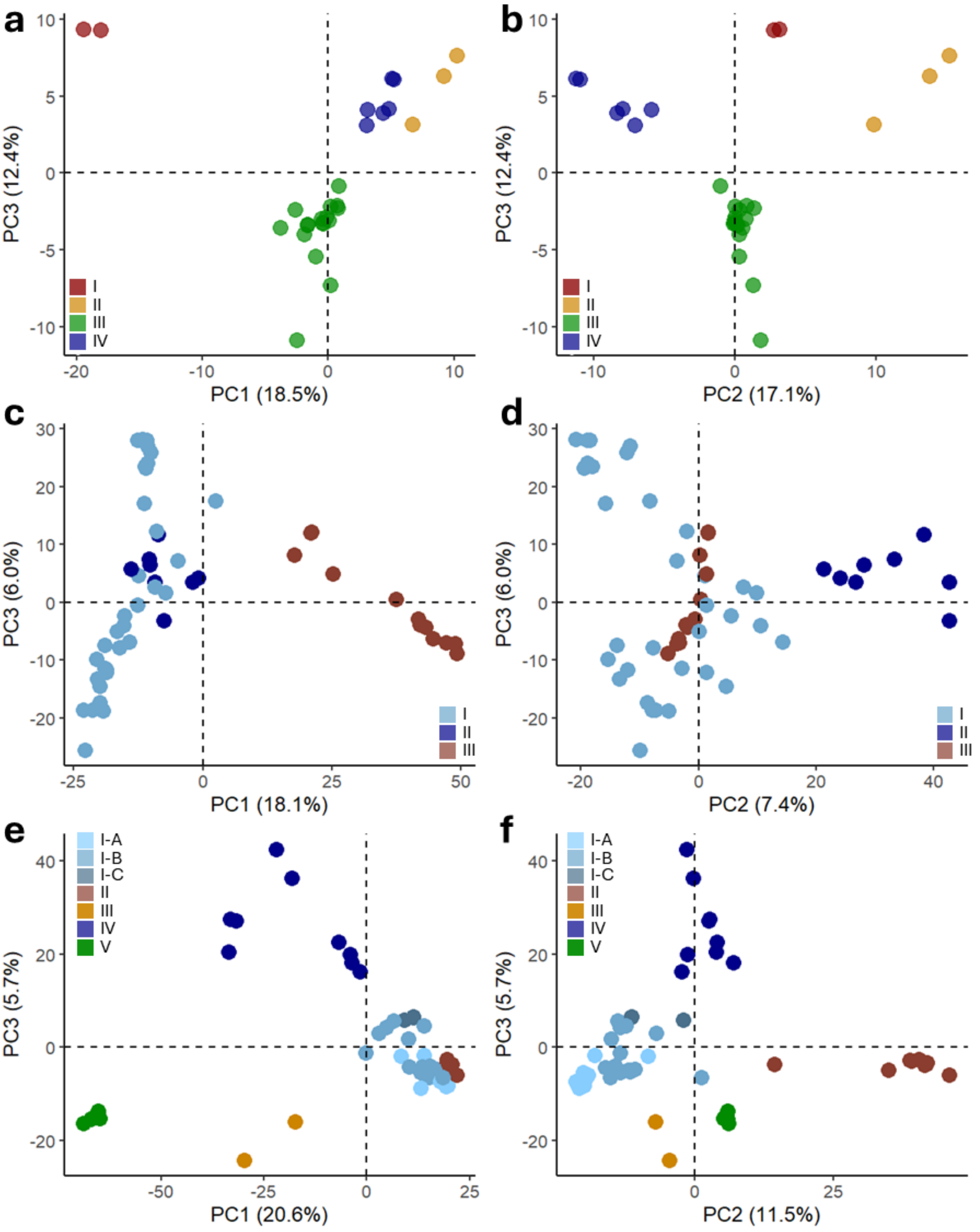


**Figure S2: PCAs of *Verticillium nonalfalfae* (a, b), *Fusarium culmorum* (c, d) and *Botrytis cinerea* (e, f).**

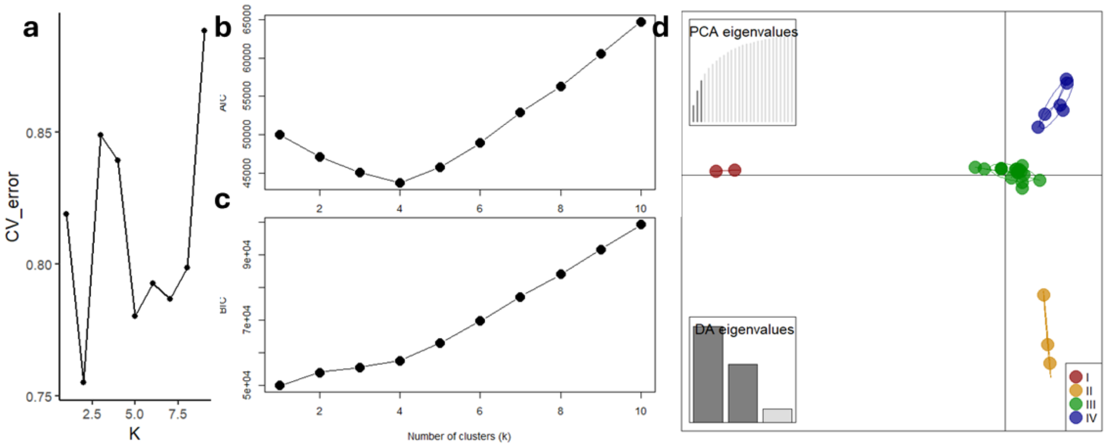


**Figure S3: *Verticillium nonalfalfae* cluster analysis**. **a**: cluster analysis from STRUCTURE, **b**: AIC model fit, **c**: BIC model fit and **d**: DAPC with 4 PCAs and 4 DAs retained.

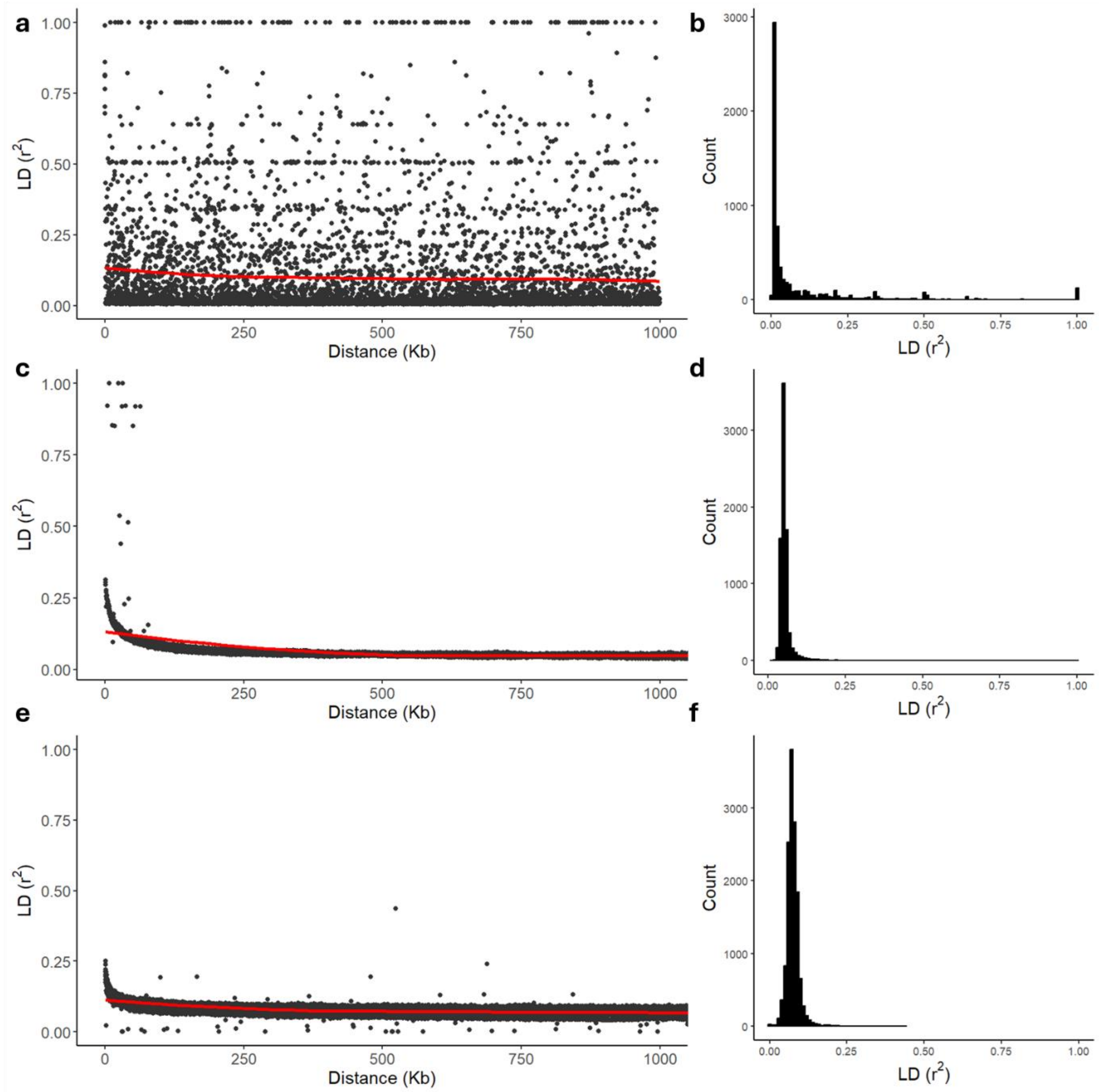


**Figure S4: Linkage disequilibrium decay analysis**. **a**: LD decay of *Verticillium nonalfalfae*, **b**: *V. nonalfalfae* count of linked SNPs, **c**: LD decay of *Fusarium culmorum*, **d**: *F. culmorum* count of linked SNPs, **e**: LD decay of *Botrytis cinerea*, **f**: B. cinerea count of linked SNPs.

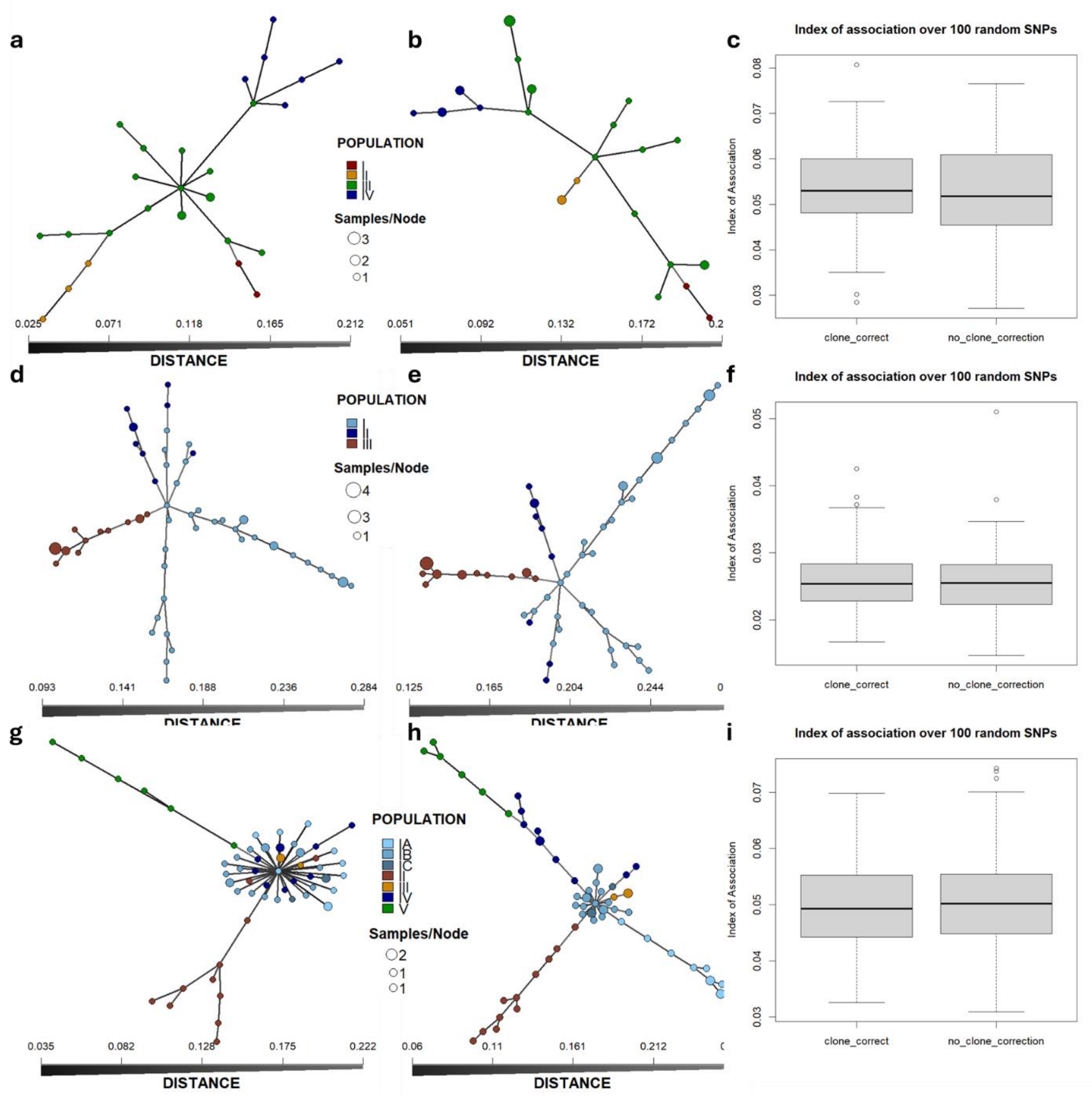


**Figure S5: Minimum spanning networks (MSNs) showing multi-locus genotypes and index of association analysis.** **a**: Not clone corrected MSN of *Verticillium nonalfalfae*, **b**: Clone corrected MSN of *V. nonalfalfae*, **c**: ia of 100 random SNPs of *V. nonalfalfae*, **d**: not clone corrected MSN of *Fusarium culmorum*, **e**: Clone corrected MSN of *F. culmorum*, **f**: ia of 100 random SNPs of *F. culmorum*, **g**: not clone corrected MSN of *Botrytis cinerea*, **h**: Clone corrected MSN of *B. cinerea*, **i**: ia of 100 random SNPs of *B. cinerea*.

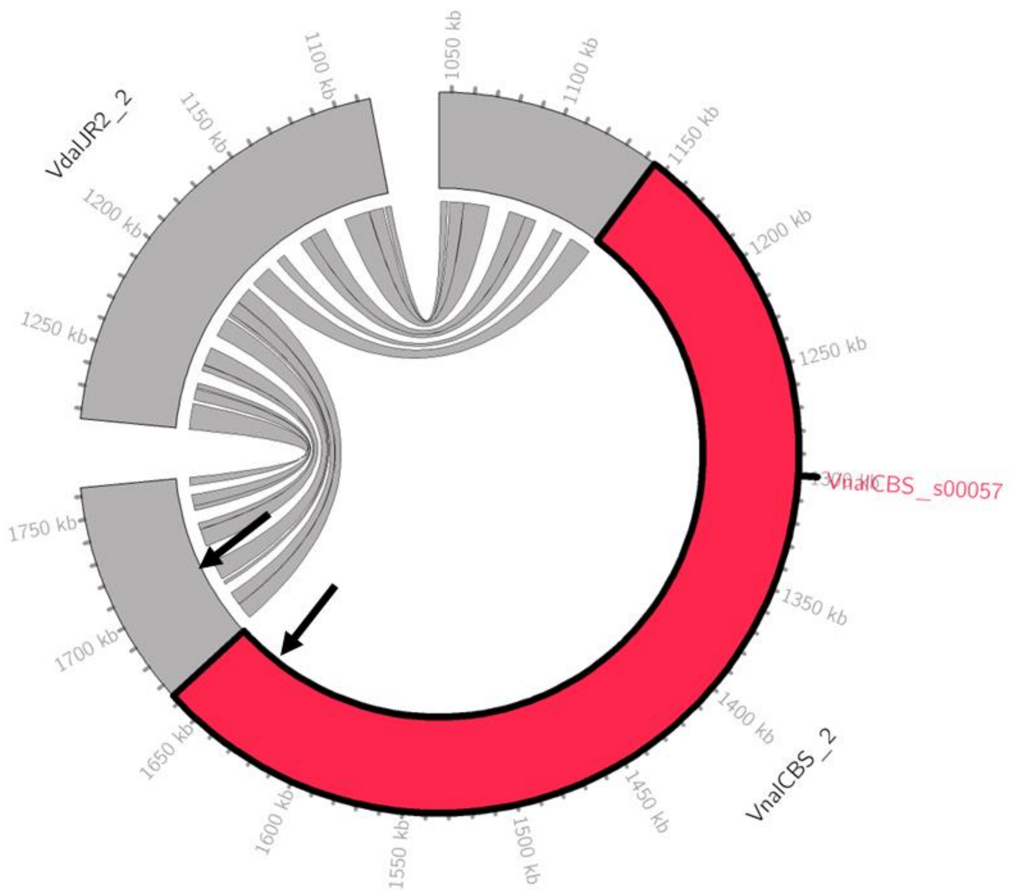


**Figure S6: Arwing family starship on chromosome 2 of Vnal01 (VnalCBS).** Confirmation was done compared to chromosome 2 of *Verticillium dahliae* strains JR2 (Vdal01). Arrows indicate SNPs associated with time (chr2_1656264, chr2_1658905 and chr2_1712892)

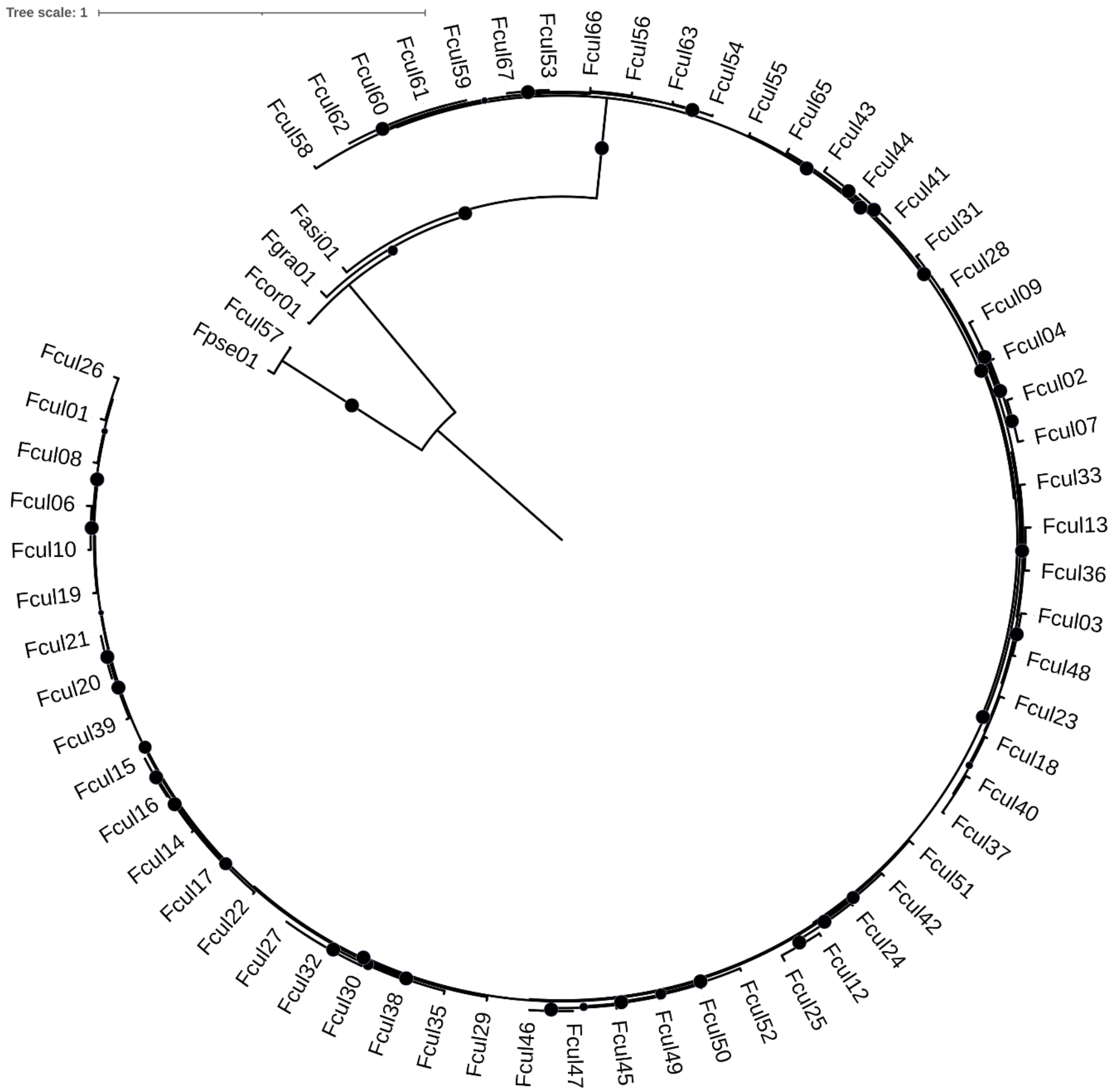


**Figure S7: *Fusarium culmorum* phylogeny.** Maximum likelihood tree from 33,768 SNPs of 67 *Fusarium* isolates. Bootstrap support values (>90) are indicated with a black dot at each node. Tree is rooted to Fpse01. Fpse = *F. pseudograminearum,* Fcor = *F. cortaderiae*, Fgra = *F. graminearum* and Fasi = *F. asiaticum.* Fcul57 is *F. pseudograminearum.* Fcul02 was used as the reference genome.

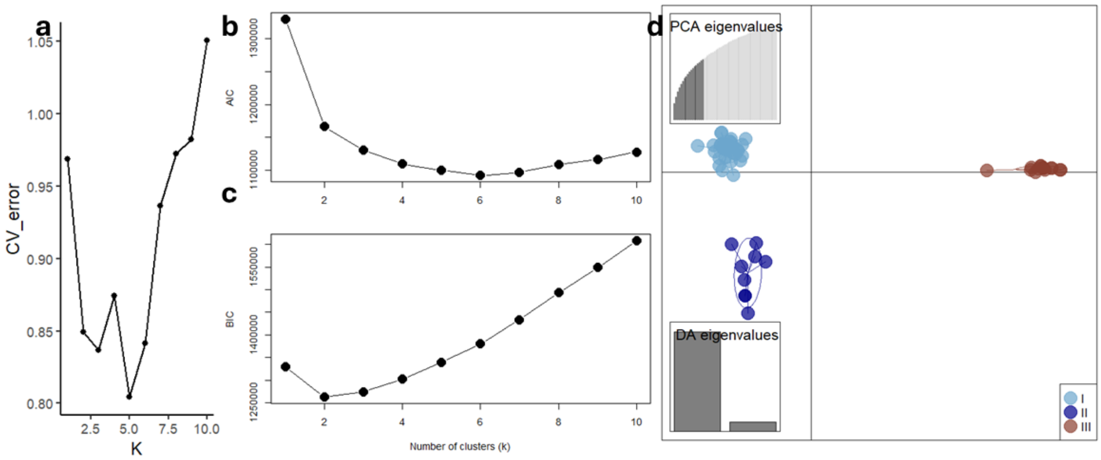


**Figure S8: *Fusarium culmorum* cluster analysis**. **a**: cluster analysis from STRUCTURE, **b**: AIC model fit, **c**: BIC model fit and **d**: DAPC with 18 PCAs and 2 DAs retained.

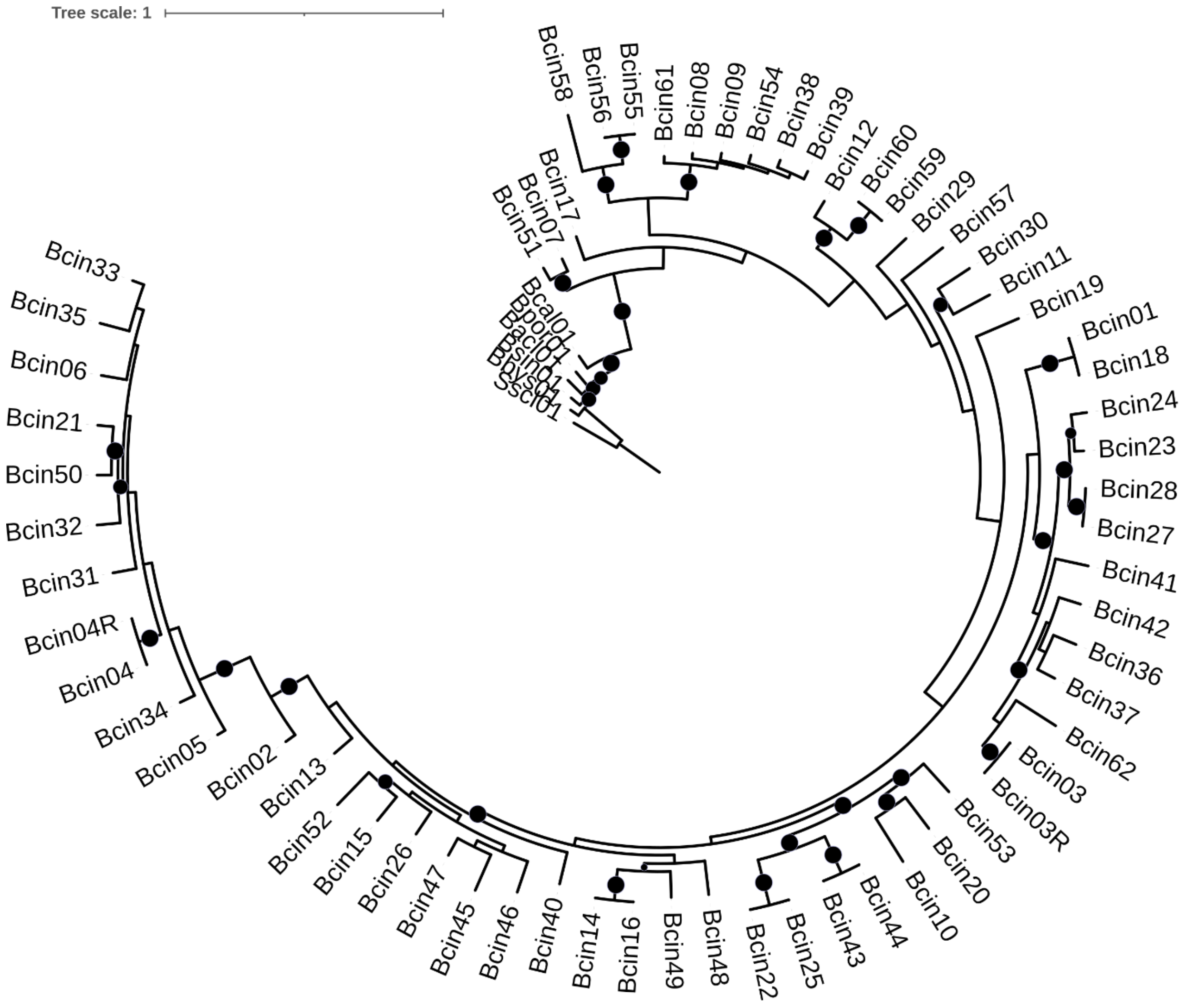


**Figure S9*: Botrytis cinerea* phylogeny.** Maximum likelihood tree from 17,655 SNPs of 70 *Botrytis* isolates. Bootstrap support values (>90) are indicated with a black dot at each node. Tree is rooted to Sscl01. Sscl = *Sclerotinia sclerotiorum,* Bbys = *B. byssoidea*, Bsin = *B. sinoalli*, Bacl = *B. aclada*, Bpor = *B. porri*, Bcal = *B. calthae* and Bcin = *B. cinerea*. Bcin04 was used as the reference genome.

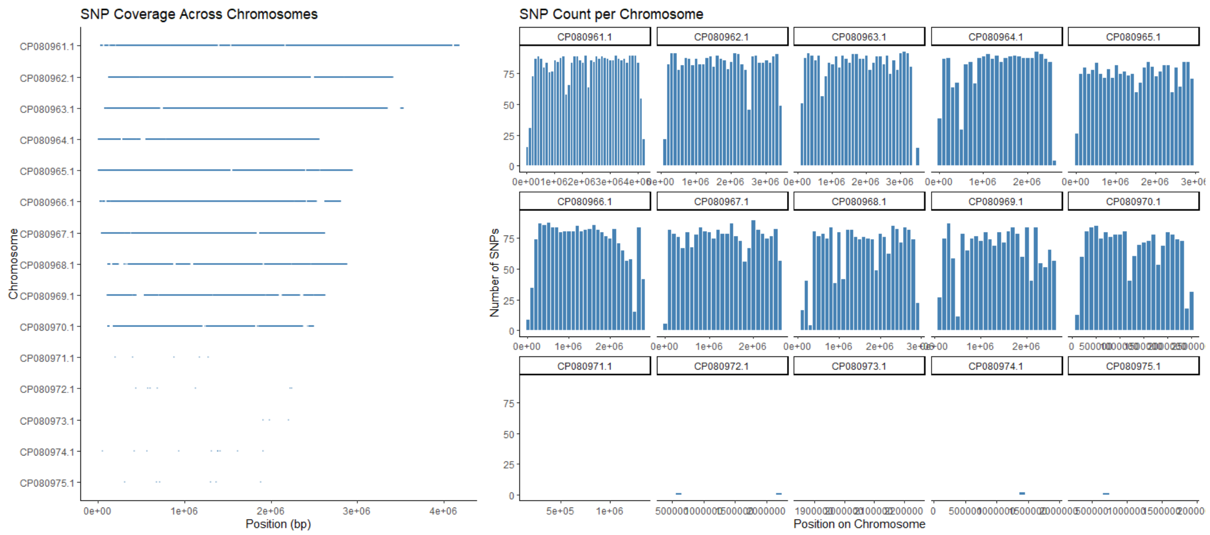


**Figure S10: SNP distribution of *Botrytis cinerea*.** Bcin04 (**Table S1**) was used as the reference genome.

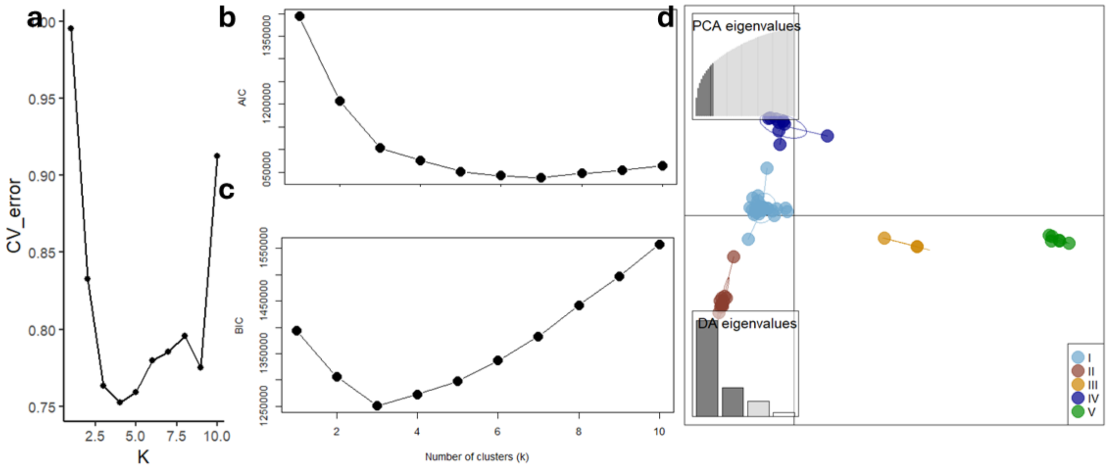


**Figure S11: *Botrytis cinerea* cluster analysis**. **a**: cluster analysis from STRUCTURE, **b**: AIC model fit, **c**: BIC model fit and **d**: DAPC with 11 PCAs and 3 DAs retained.
